# Hyperhemolytic Crisis Following Transfusion in Sickle Cell Disease With Acute Hepatic Crisis: A Case Report

**DOI:** 10.7759/cureus.27844

**Published:** 2022-08-10

**Authors:** Krunal Trivedi, Ahmed Abbas, Riyashat Kazmi, Hamid Shaaban, Richard Miller

**Affiliations:** 1 Internal Medicine, Saint Michael's Medical Center, Newark, USA; 2 Hematology/Oncology, Saint Michael's Medical Center, Newark, USA; 3 Pulmonary and Critical Care Medicine, Saint Michael's Medical Center, Newark, USA

**Keywords:** alloimmunization, transfusion related complications, hepatic crisis, hyperhemolytic crisis, sickle cell disease

## Abstract

Hyperhemolysis syndrome (HHS) is a catastrophic unpredictable consequence of blood transfusion in sickle cell disease. It leads to further drop in hemoglobin via immune mechanisms complicating a hospital course and prolonging length of stay. Although sickle cell patients receive multiple transfusions throughout their disease course, this condition remains underreported by health care professionals or misinterpreted for other sickle cell crises. We present a similar case highlighting the importance of early recognition of HHS and judicious blood transfusion in sickle cell disease patients to avoid such a complication.

## Introduction

Hyperhemolysis syndrome (HHS) is a rare complication of repeated blood transfusions in sickle cell disease that is often unrecognized due to its rapid progression and similarity to other complications of sickle cell disease [1]. To date, no literature clearly establishes the incidence of HHS in sickle cell disease (SCD). Patients with SCD often present with low hemoglobin. Subsequent transfusions may cause hyperhemolysis syndrome, whereby both native and donor red blood cells undergo further hemolysis, leading to lower post-transfusion hemoglobin levels. It can present acutely within seven days of the transfusion incident or may be delayed whereby alloantibodies are more likely to have formed [2]. We report a case of HHS in a patient who was initially admitted for a sickle cell painful crisis, developed signs of hemolysis acutely after he got blood transfusion to highlight the importance of early recognition of such a rare phenomenon besides being hypervigilant to a patient's blood transfusion record.

## Case presentation

A 23-year-old male patient with past medical history of sickle cell disease and questionable lupus presented to the emergency department for one-day history of bilateral chest, lower back, and bilateral hip pain, rated 10/10 in severity, not amenable to analgesics. He was admitted for acute painful crisis six months ago, having received IV fluids and opioids. He was discharged on hydroxyurea and folate. He failed to follow up with his hematologist and had not refilled his hydroxyurea the month prior to his symptoms. He actively engaged in receptive anal intercourse with the same male partner and developed bleeding per rectum the week before. Review of systems was unremarkable for fever, chills, cough, nausea, vomiting, diarrhea, or skin rash. Physical examination was remarkable for conjunctival pallor and mild-scleral icterus. Initial labs were as shown in Table 1.

**Table 1:** Initial laboratory results of the patient. Hb: hemoglobin; MCV: mean corpuscle volume; MCH: mean corpuscular hemoglobin; MCHC: mean corpuscular hemoglobin concentration; WBC: white blood cells; LDH: lactate dehydrogenase; ALT: alanine transaminase; AST: aspartate transaminase; ALP: alkaline phosphatase

Laboratory parameters	Value	Reference range
Hb	8.8 g/dL (at baseline)	13.5-17.5 g/dL
MCV	70.6 fL	81-95 fL
MCH	24 pg	27.5-33 pg
MCHC	34 g/dL	33.5-35.5 g/dL
WBC	14.3x10³/L	4-11x10³/L
LDH	1800 U/L (peaked at 2600)	122-222 U/L
Bilirubin total	4.2 mg/dL (peaked at 7)	0.2-1.2 mg/dL
Bilirubin direct	2.77 mg/dL	0-0.3 mg/dL
Haptoglobin	<31 mg/dL	30-200 mg/dL
Reticulocyte	3.8%	0.5-1.5%
ALT	70 U/L (peaked at 1200)	9-46 U/L
AST	140 U/L (peaked at 1600)	10-36 U/L
ALP	140 U/L (at baseline)	40-115 U/L

Patient was initially admitted and placed on IV fluids, opioids, and empiric antibiotics for acute chest syndrome. He was then upgraded to the intensive care unit for change in mental status, persistent sinus tachycardia of 140 beats per minute, and acute drop in hemoglobin (Hb). One unit packed red blood cells (PRBC) was ordered with further drop of Hb to 5.5 g/dL.

In view of worsening mental status and concern for acute stroke, two additional units of PRBCs were administered. Head computed tomography was normal. A repeat workup showed resolving hemolysis, hence no steroids or IV immunoglobulins were given. Mental status gradually improved and the patient was downgraded to floors.

**Figure 1:**
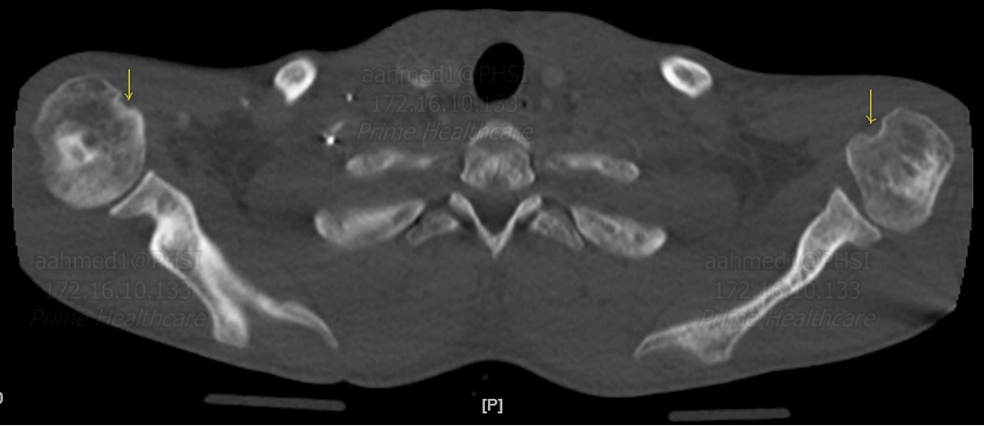
CTA chest axial bone window showing bilateral erosions and collapse of humeral heads consistent with avascular necrosis. CTA: CT angiography

**Figure 2:**
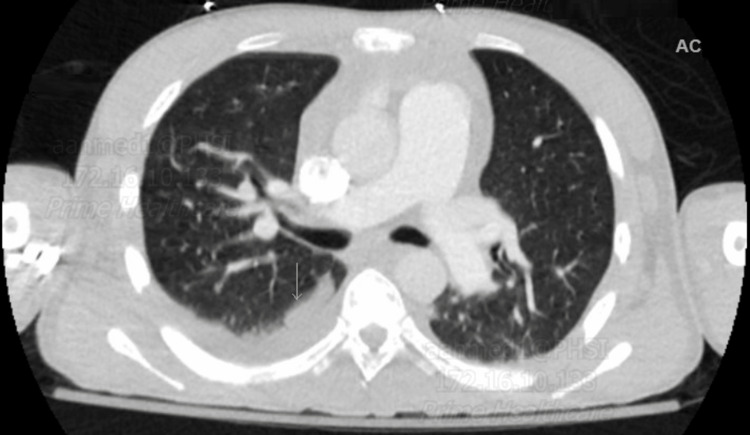
CTA chest axial lung window showing right lower pleural effusion with possible atelectasis and consolidation. CTA: CT angiography

**Figure 3:**
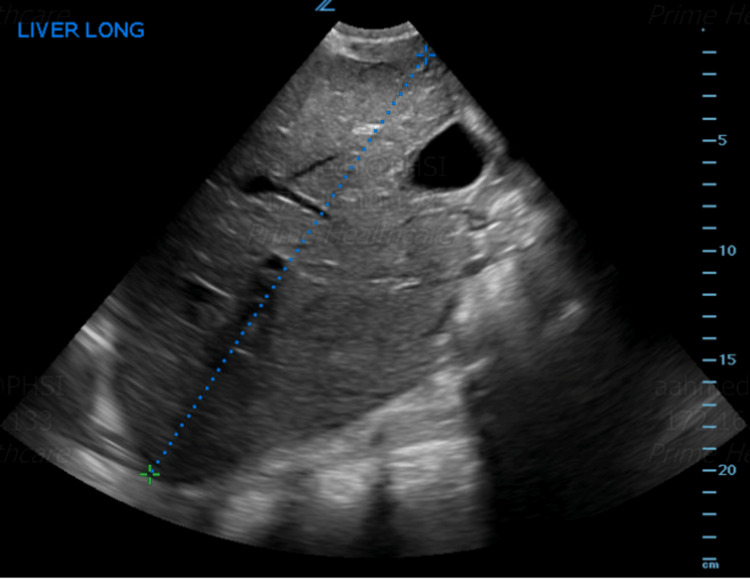
US abdomen showing hepatomegaly measuring 22.8 cm in longitudinal dimension.

Chest CT angiography ruled out pulmonary embolism but showed interval avascular necrosis of bilateral humeral heads, right pleural effusion with adjacent atelectasis, and possible lower lobe consolidation (Figures 1, 2). Incentive spirometry was employed. The ultrasound abdomen showed hepatomegaly consistent with sickle cell hepatopathy (Figure 3).

Iron Panel test, vitamin B 12 level, fibrinogen, gonococcus/chlamydia nucleic acid amplification, HIV antibodies, parvovirus B-19 immunoglobulin M, and blood cultures were all normal. Peripheral smear showed microcytic anemia with marked anisopoikilocytosis, sickle cells, target cells, and nucleated RBCs consistent with SCD. 

The patient had no new complaints, bilirubin trended down, hematology weaned him off hydromorphone, and he was discharged on hydroxyurea, folic acid, and as-needed Percocet for one week.

## Discussion

Hyperhemolysis syndrome is more prevalent in patients with underlying hemoglobinopathies, such as thalassemia, hemoglobin C, or hemoglobin SC, or hematologic disorders such as myelofibrosis, anemia of chronic disease, and lymphoma. However, SCD presents the most documented cases [3].

SCD patients are more likely to develop alloantibodies with HHS and other hemolytic reactions for the following multiple reasons: (1) lifelong blood transfusions for symptomatic anemia or acute crises, (2) receiving blood products from predominantly Caucasian donors, (3) gene-related factors, and (4) inability to clear transfused Hb completely owing to defective reticuloendothelial system, resulting in an inflammatory state with increased risk of alloimmunization [4,5].

**Figure 4:**
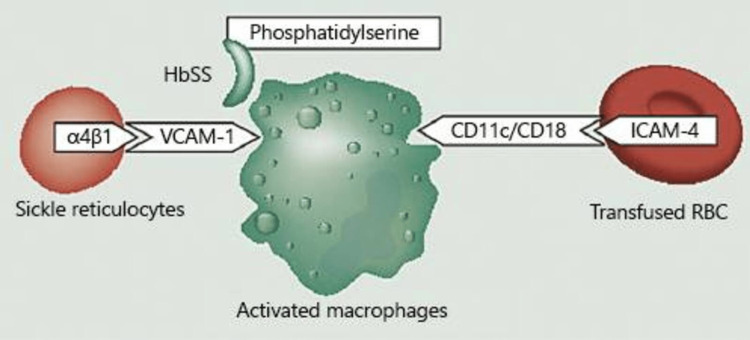
Destruction of native and transfused red cells by activated macrophages due to exposure to phosphatidylserine. HbSS: sickle cell hemoglobin; α4β1: hemoglobin subunits in a sickle cell; VCAM-1: vascular cell adhesion molecule-1; ICAM-4: intercellular adhesion molecule-4; CD: cluster of differentiation The figure is obtained with permission from Madu et al. (2021) [7].

Many theories try to explain how HHS develops in SCD, including "RBC phosphatidylserine exposure," "bystander complement activation," and "suppressed erythropoiesis." Sickled RBCs (owing to osmotic damage, oxidative stress, or ATP depletion) express more phosphatidylserine on their surface, which marks them for clearance by the activated macrophage [6,7]. Bystander hemolysis occurs when both native and donor RBCs are hemolyzed leading to a lower post-transfusion hemoglobin. Antibodies against donor RBCs are believed to activate bystander complement to destroy native RBCs with reticulocyte precursors [7,8]. HHS is thought to work through multiple mechanisms because of its consumptive effect (Figure 4).

HHS is diagnosed when an SCD patient develops worsening anemia, fever, and jaundice following PRBC transfusion (usually in the setting of acute crisis). Important lab findings include elevated lactate dehydrogenase, elevated bilirubin, decreased reticulocyte count, and unchanged urine HbA/HbS ratio as lysis affects both donor and native RBC (the ratio is decreased in delayed hemolytic transfusion reaction {DHTR} as lysis only affects donor RBCs).

There are two forms of HHS - acute and delayed. In the acute form, symptoms are seen within seven days of the transfusion and alloantibodies do not typically form within this time period, so direct antiglobulin test (DAT) would be negative. If the patient shows symptoms beyond seven days after transfusion, alloantibodies have more likely formed and DAT would be positive. Our patient showed symptoms within seven days of transfusion, with positive hemolytic workup and DAT came back negative [6].

Treatment of HHS is to stop all blood transfusions and carefully consider the necessity of future transfusions [8]. In situations that require transfusions, intravenous immunoglobulin G (IVIG) and corticosteroids may be considered [6,9]. Currently, it is recommended to give IVIG in low dose (0.4 g/kg/day) for five days and methylprednisolone in low dose (0.5 g/day for adults and 4.0 mg/kg/day for children) for two days [8]. Because the reticulocyte count is rapidly restored by rituximab, this can be an effective treatment [10]. A potential treatment, erythropoietin, has been investigated in order to overcome erythropoiesis suppression, however, more research is needed to prove its effectiveness. Similarly, eculizumab, a C5 convertase inhibitor, inhibits the complement cascade and aids in the maintenance of RBC membrane integrity in patients with SCD [11]. In the treatment of severe hemolysis and severe hyperhemolysis, therapeutic plasma exchange has shown to be successful [12]. Hemoglobin-based oxygen carrier substitutes, such as cross-linked tetrameric hemoglobin and conjugated tetrameric hemoglobin, may be beneficial as oxygen carriers and for their potential to reduce free Hb S, an oxidizer, and proinflammatory agent. A study is currently needed because the US FDA has not yet approved any hemoglobin-based oxygen carrier substitutes [13].

Acute hepatic crisis has been observed in approximately 10% of adults with SCD [14]. There are very few reports of hepatic crises, mostly as isolated cases [15]. Although its etiology is unclear, the sickled erythrocytes are believed to adhere to the hepatic vascular endothelium, causing congestion, tissue infarction, and in more severe cases, liver dysfunction [16]. Patients typically present with acute pain in their right upper abdominal quadrant, nausea, low-grade fever, tender hepatomegaly, as well as yellowing of the skin. The serum alanine and aspartate aminotransferase are seldom >300 IU/L. Aspartate aminotransferase is also elevated by hemolysis, so serum alanine aminotransferase may be a more accurate indicator of liver injury [17].

Rapid reduction of Hb S by exchange transfusion (ET) is the cornerstone of the treatment in acute hepatic crisis. Supportive treatments include hydration with intravenous fluid, analgesia, correction of coagulopathy, and monitoring electrolytes [18]. In patients with full-blown liver failure, liver transplantation has been proposed as a treatment option. Transplantation has, however, not been widely used [19].

## Conclusions

Although sickle cell patients receive multiple blood transfusions throughout their disease course, hyperhemolysis syndrome remains underreported by health care professionals. Conservative approach to blood transfusion in sickle cell disease remains crucial to avoid transfusion-related complications and reduce the chance of alloimmunization when blood is needed. A high index of suspicion for hyperhemolysis syndrome and identification of the transfusion records is the way to early intervention.
